# Prevalence of undernutrition among pregnant women and its differences across relevant subgroups in rural Ethiopia: a community-based cross-sectional study

**DOI:** 10.1186/s41043-023-00358-6

**Published:** 2023-03-10

**Authors:** Nana Chea, Yadessa Tegene, Ayalew Astatkie, Mark Spigt

**Affiliations:** 1 School of Public Health, College of Medicine and Health Sciences, Hawassa University, P.O.Box: 1560, Hawassa, Ethiopia; 2 School CAPHRI, Care and Public Health Research Institute, Maastricht University, Maastricht, The Netherlands; 3 General Practice Research Unit, Department of Community Medicine, The Arctic University of Tromsø, Tromsø, Norway

**Keywords:** Undernutrition, Food taboo, Nutritional counseling, Miscarriage, Pregnant women, Sidama, Ethiopia

## Abstract

**Background:**

Maternal undernutrition is one of the commonest public health problems in many low- and middle-income countries where generally more than 20% of women are undernourished. It is more common in rural areas due to unclear factors. Therefore, the aim of this study was to investigate the prevalence of undernutrition in general and in subgroups and determine risk factors among pregnant women in rural Ethiopia.

**Methods:**

A community-based cross-sectional survey was conducted from April 30 to May 30, 2019 on 550 pregnant women who were randomly selected from six districts in southern Ethiopia. Trained and experienced nurses measured undernutrition using mid-upper arm circumference and collected other data. We used multilevel mixed-effect logistic regression to identify factors associated with undernutrition among pregnant women.

**Results:**

The prevalence of undernutrition among pregnant women was 38% (95% CI: (34.2–42.3). The odds of undernutrition was higher among women who got pregnant previously (adjusted odds ratio [AOR]: 1.66; 95% CI: 1.02–2.71), who had a history of miscarriage (AOR: 3.18; 95% CI: 1.77–5.70), who practiced food taboos (AOR: 2.23; 95% CI: 1.47–3.39), and who did not get any nutritional counseling during pregnancy (AOR: 2.97; 95% CI: 1.79- 4.95). The prevalence of undernutrition was higher among pregnant women who had multiple risk factors and the difference was statistically significant (*p* < 0.001).

**Conclusion:**

Undernutrition is a highly prevalent problem among rural Ethiopian pregnant women, especially with those who avoid food, do not get counseled, and had two or more pregnancies and a history of miscarriage. Improving the integration of nutrition programs with routine healthcare services and encouraging a multi-sectorial intervention strategy would help to reduce maternal undernutrition in the country.

## Key message

- Undernutrition is a highly prevalent problem among rural Ethiopian pregnant women, especially with those who avoid food/s, do not get counseled, and had two or more pregnancies and a history of miscarriage.
- All healthcare providers, especially those working in ANC and child services in Ethiopia, should be aware of the risks of maternal undernutrition.
- Nutritional education and counseling at the ANC clinic should be strengthened, and there should be reinforcement mechanisms

## Background

Globally, the burden of maternal undernutrition remains a huge public health problem, with around 10% of women being undernourished [1]. A systematic review from Africa revealed that the overall pooled prevalence of maternal undernutrition was 20% [2]. However, the prevalence of undernutrition in different regions of Africa seems to vary considerably. A study from Kenya, for example, found more than half (60%) of women to be moderately to severely malnourished, and the same proportion of women suffered from anemia [3]. In the Ashanti region of Ghana, the overall prevalence of anemia among pregnant women was 56% [4]. In Ethiopia, though maternal undernutrition decreased from 30% in 2000 to 22% in 2016, undernutrition among pregnant women remained as one of the most important public health problems, with prevalence estimates ranging from 14 to 45%. [5–9] A study indicated that maternal undernutrition was 1.5 times higher in rural than urban areas. [10]

Undernourished women are at increased risk of having low-birth weight infants, which will result in a weaker developmental path for the child, both physically and mentally [7]. Preterm birth, intrauterine growth restriction, and reproductive loss through stillbirths are also often a result of maternal undernutrition [11]. Moreover, 20% of maternal deaths is attributed to maternal undernutrition [12]. Recent studies suggest that good nutrition during pregnancy and breast-feeding may also reduce the risk of chronic diseases of the child later in life. [13, 14]

Studies pointed out that multiple factors affected maternal undernutrition. Maternal age, type of food consumed, foods preference, and abstaining from some nutritious food seem to be associated with maternal undernutrition during pregnancy [15–23]. Furthermore, the presence of acute or chronic infection, inadequate food intake, educational status, income, and other factors were associated with maternal undernutrition in previous studies. [2, 5–8, 24]

Local circumstances in different regions may differ, necessitating intervention targeting specific subgroups of pregnant women. Providing interventions for all women, including those with a low risk of undernutrition, might not be very efficient. We were interested in whether it was possible to identify subgroups with a higher risk of undernutrition and in identifying the underlying reasons, for this higher prevalence. Specifically to the study area, it seems that pregnant women avoid some foods during pregnancy for unknown reasons and we wanted to know whether this tradition was associated with the prevalence of undernutrition [23, 25, 26]. Therefore, the aim of this study was to assess the prevalence of undernutrition among pregnant women in general and identify subgroups with a high risk of undernutrition.

## Methods

### Study design

A community-based cross-sectional study was carried out from April 30 to May 30, 2019 among pregnant women in six randomly selected districts of Sidama Region, Southern Ethiopia.

### Study area

Sidama National Regional State is one of the 10 regional states of Ethiopia. Hawassa City is the capital city of the region and is located around 273 KM south of Addis Ababa, the capital city of the country. Its geographic location lies between 4027’and 8030’latitude north and 340 21’and 39,011’longitude east. The total area of the region is about 6981.8 Km^2^ and is divided into 30 districts**.** As reported by the regional health bureau, the region has 1 referral, 4 general, and 13 primary hospitals, and 137 health centers. The region’s population accounts for 4.0% of the national population, and nearly 23% and 3.5% of the population are expected to be reproductive age groups and pregnant women, respectively.

### Sample size and sampling procedures

A single population proportion formula was used to calculate the sample size. A prevalence of 31.8% was taken as a prior estimate from a study conducted on undernutrition among pregnant women in the central Rift Valley of Ethiopia, in southern Ethiopia [7]. A 95% level of confidence and a 5% margin of error to be tolerated were considered. This resulted in a required sample of 333 women, which was subsequently multiplied by 1.5 for design effect and adjusted for a 10% non-response rate. Finally, the sample size considered was 550.

We used a multi-stage sampling technique to select eligible pregnant women. First, six districts were selected randomly out of the 21 districts of the Sidama region. In stage two, 18 ‘*kebeles*’ (the smallest administration units in Ethiopia) were selected using simple random sampling. In stage three, 550 pregnant women were selected using simple random sampling techniques. The registration book of the Health Extension Worker (community health worker in Ethiopia) for pregnant women was used to select study participants as the sampling frame. Finally, selected pregnant women from each village were interviewed at their homes. Pregnant women who were registered as residents for less than six months were considered not representative of the *kebeles* population and were therefore excluded. Also excluded were women who would be unable to give answers in an interview, such as those having hearing or speech problems, or who should not be bothered with an interview, for example, due to severe hyperemesis.

### Variables of the study

#### Dependent variable

Undernutrition in pregnant women was the dependent variable. It was measured using a mid-upper arm circumference (MUAC) measuring tape to the nearest 1 mm at the mid-point between the shoulder and elbow on the woman's bare left arm. In the literature, there is inconsistency in the use of the MUAC cutoff value for pregnant women. For instance, previous studies on similar populations in the country used different MUAC cutoff points [13, 27]. However, based on current literature in different corners of the country, we defined undernutrition using a MUAC value of less than or equal to 23 cm. It was dichotomized as “normal” (MUAC > 23) and “undernutrition” (MUAC ≤ 23). [6, 16]

#### Independent variables

Independent variables were grouped into individual and community level variables.

##### Individual level variables

In this study, women's socio-demographic characteristics (age, education, income, and occupation), reproductive health-related characteristics (ANC follow-up, gestational age of pregnancy, gravidity, use of family planning, miscarriage), and variables related to foods and nutrition like food taboos (avoiding one or more specific foods during pregnancy) and reasons for it were all considered individual-level variables.

##### Community level variables

Community level variables include kebele of residence and respondent districts, which were contextualized as “Dega” (cool zone), which is above 2440 m in elevation (Hula and Bona districts), “Woinadega” (subtropical zone), which includes the highlands areas of 1830–2440 m in elevation (Loka Abaya), and “kola” (tropical zone), which is below 1830 m in elevation (Aleta Wondo).

### Data collection instrument and procedure

A structured questionnaire was used to collect the data. The questionnaire was initially prepared in English, translated into Sidaamu Afoo, and then translated back to English to check its consistency before administration. The questionnaire, which was designed to generate data related to the dependent and all independent variables, was pretested, and amendments were made accordingly. Pregnant women were invited for an interview at home. Potential respondents who were busy or unavailable on the date of data collection were revisited three times before being deemed non-respondents. Six Bachelor of Science (BSc) holders in nursing profession, who had experience with anthropometric measurements and were fluent in the local language, collected both the questionnaire and anthropometric data. The principal investigator and two recruited field supervisors supervised the overall data collection activity. The data collectors and supervisors were trained for one day about the techniques of data collection, and anthropometric measurement, and other issues of the research.

### Data analysis

We entered data into the Epi-data entry software (version 3.1). Further data cleaning and descriptive analysis were done using the Statistical Package for the Social Sciences (SPSS) version 24. Since we collected data from multi-stage sampled units and our data were hierarchical in nature, that is, women are nested within households, households are nested within kebeles, and kebeles are nested within districts, analyzing this data using ordinary logistic regression analysis could poorly estimate the standard error of effect size. In another word, women within a kebeles may be more similar to each other than women in the rest of the kebeles. Similarly, women within a district may be more similar to each other than women in the rest of the districts. To identify factors associated with undernutrition among pregnant women, we used a multilevel mixed-effect logistic regression model and data were analyzed using Stata Statistical Software version 14.0 (StataCorp College Station, TX, USA). The model employed the robust standard-error estimator and the main effect of independent variables. We checked for multicollinearity using the variance inflation factor (VIF), which was nearly 1%. To assess model fitness, we used Akaike’s information criterion (AIC) [30], and for the measure of variation (random effect), the median odds ratio (MOR) and intra-class correlation coefficient (ICC) were computed. Accordingly, a mixed-effect logistic regression model (both fixed and random effects) was the best-fitting model since it had the lowest deviance value. Descriptive analysis was done to measure the proportion of socio-demographic and other characteristics of pregnant women and the prevalence of undernutrition. A bivariate analysis of the association between independent variables and maternal undernutrition was performed to identify factors for inclusion into the multivariate model. Accordingly, variables with a p value < 0.20 [31], and three important socio-demographic variables such as age, educational status, and income, though their p values > 0.20, were considered in the multivariate multilevel analysis to control for cofounders. An adjusted odds ratio (AOR) with a 95% confidence interval was reported. A variable with an AOR not including 1 and p value of less than 0.05 was used to declare the presence of statistical significance.

## Results

### Socio-demographic characteristics of study participants

From the total random sample of 550, twelve women refused to participate, and eight women were excluded due to severe hyperemesis. We had complete data from 530 women, resulting in a response rate of 96%. The mean (± Standard Deviation [SD]) age of the respondents was 27 ± 4.9 years and ranging from 18 to 42 years**.** Over half of the women (52%) had no formal education, and half (50%) were housewives by occupation. At the time of the study, only 1% of the women had a monthly income of more than 1000 Ethiopian Birr (ETB) (equivalent to 21 United States Dollars [USD]). All participants were married, and 10% of them were in polygamous marriages (Table 1).

**Table 1:** Socio-demographic characteristics of the study participants, Sidama, Ethiopia, June, 2019

Variables	Nutritional status of pregnant women		COR (95% CI)	AOR (95% CI)
	Normal	Undernutrition		
*Age*				
18–24	104 (66%)	53 (34%)	0.69 (0.35–1.99)	0.99 (0.46, 2.14)
25–34	195 (61%)	126 (39%)	0.88 (0.66–1.18)	0.89 (0.32–1.43)
> 35	30 (58%)	22 (42%)	1	1
*Occupation*				
House wife	159 (60%	106 (40%)	1	
Merchant	114 (66%)	58 (34%)	0.76 (0.51–1.15)	
employee	56 (60%)	37 (40%)	0.99 (0.54–1.82)	
*Religion*				
Christian	321 (62%)	197 (38%)	1	
Muslim	8 (67%)	4 (33%)	0.81 (0.39–1.71)	
*Ethnicity*				
Sidama	303 (62%)	189 (38%)	1	
Amhara	17 (65%)	9 (35%)	0.85 (0.36–2.00)	
Other	9 (75%)	3 (25%)	0.53 (0.14–1.99)	
*Marriage type*				
Monogamy	299 (63%)	177 (37%)	1	
polygamy	30 (56%)	24 (44%)	1.35 (0.76–2.38)	
*Education*				
no formal education	170 (62%)	105 (38%)	0.83 (0.39–1.81)	0.71 (0.33–1.54)
Primary education	136 (63%)	79 (37%)	0.78 (0.41–1.51)	0.67(0.32–1.43)
Secondary and higher education	23 (57%)	17 (43%)	1	1
*Average monthly income*				
< 500 ETB	285 (62%)	174 (38%)	0.81 (0.15–4.33)	0.79(0.15–4.06)
501-1000ETB	40 (62%)	24 (38%)	.80 (0.14–4.59)	0.87 (0.16–4.88)
> 1000ETB	4 (57%)	3 (63%)	1	1

### Reproductive health aspects

Almost two-thirds (69%) of the study participants already gave birth to a baby before, 57% were in the third trimester, and 41% had no ANC follow-up for the index child. Most (76%) of the study participants used family planning methods regularly. Thirteen percent of the women had a history of miscarriage (Table 2).

**Table 2:** Reproductive health characteristics of the study participants, Sidama, Ethiopia, June, 2019

Variables	Nutritional status		COR (95% CI)	AOR (95% CI)
	Normal	Undernutrition		
*Gestational age*				
Second trimester	147 (64%)	83 (36%)	0.87 (0.58–1.31)	
Third trimester	182 (61%)	118 (39%)	1	
*ANC Follow up*				
Yes	195 (62%)	118 (38%)	1	
No	134 (62%)	83 (38%)	1.02 (0.51–2.04)	
*Family planning*				
Yes	253 (63%)	151 (37%)	1	
No	76 (60%)	50 (40%)	1.10 (0.81–1.49)	
*History of miscarriage*				
Yes	24 (34%)	46 (66%)	3.83 (2.46–5.96)*	3.18 (1.77–5.70)**
No	305 (66%)	155 (34%)	1	1
*Birth spacing*				
19 and more month	188 (68%)	89 (32%)	1	1
18 and less months	24 (27%)	65 (73%)	5.72 (4.06–8.05)*	
*Gravidity*				
Prim gravidity	116 (71%)	48 (29%)	1	1
Multi gravidity	213 (58%)	153 (42%)	1.74 (1.17–2.57)*	1.66 (1.02–2.71)**

*indicates that the variable has association

**indicates that the variable was statistically significant at *P* value ≤ 0.05

### Nutrition and feeding characteristics of study participants

Of the total 530 women, 38% (95% CI; 34.2–42.3) had a MUAC of ≤ 23 cm indicating undernutrition. Thirty-two percent (32%) of the study participants practiced food taboos during pregnancy. Foods commonly avoided were eggs, butter, meat, and avocado. Seventy-six women (44%) reported fear of having to deliver a big or fat baby as a reason to avoid such foods. Additionally, 50% of the women increased their meal eating pattern (frequency) during pregnancy. Furthermore, 58% of the study participants received nutritional counseling during pregnancy (Table 3).

**Table 3:** Food and feeding characteristics of the study participants, Sidama, Ethiopia, June, 2019

Variables	Nutritional status		COR (95% CI)	AOR (95% CI)
	Normal	Undernutrition		
*Food taboos practice*				
No	252 (70%)	106 (30%)	1	1
Yes	77 (45%)	95 (55%)	2.93 (1.46–5.85)*	2.23 (1.47–3.39)**
*Foods commonly avoided*				
Egg	30 (52%)	28 (48%)	0.37 (0.06–2.08)	
Butter	20 (48%)	22 (52%)	0.44 (0.08–2.53)	
Meat	11 (32%)	23 (68%)	0.84 (0.14–5.01)	
Avocado	14 (45%)	17 (55%)	0.48 (0.08–2.89)	
other	2 (29%)	5 (71%)	1	
*Common reasons for food taboos*				
Fear to fat baby to delivery	33 (43%)	43 (57%)	0.43 (0.11–1.73)	
Fear of fetal abnormality	30 (57%)	23 (43%)	0.26 (0.06–1.05)	
Fear of miscarriage	14 (41%)	20 (59%)	0.48 (0.11–2.08)	
Other	3 (25%)	9 (75%)	1	
*Additional meal*				
Yes	178 (67%)	88 (33%)	1	1
No	151 (57%)	113 (43%)	1.51 (1.21–1.88)*	0.69 (0.42–1.14)
*Nutritional counseling*				
Yes	223 (73%)	83 (27%)	1	1
No	106 (47%)	118 (53%)	3.00 (2.08–4.33)*	2.97 (1.79–4.95)**

*indicates that the variable has association

**indicates that the variable was statistically significant at *P* value ≤ 0.05

### Risk factors for undernutrition among pregnant women

Based on the smallest value of deviance (reduced from 93,364.04 to 92,235.6), we understood that the mixed-effects logistic regression model was the best-fitting model for our data. Furthermore, the ICC value in the null model was 5.9% (95% CI: 3.2%, 8.7%) and reduced to 4.1% (95% CI: 2.2%, 5.9%) in the multivariable model, indicating that about 4.1% of the total variability of the undernutrition among pregnant women was attributed to district variability. The remaining 95.9% of the total variability was accounted for by the between-individual variation. The MOD was 1.2 (0.8, 1.5) and indicated that if two pregnant women were selected randomly from different districts, a pregnant woman from a district with high risk of undernutrition was 1.2 times more likely to be undernourished than a pregnant woman from a district with a lower risk of undernutrition.

Under multilevel mixed-effects-bivariate logistic regression analysis, the prevalence of undernutrition was higher among women who had a short inter-pregnancy interval, had two or more pregnancies, had a history of miscarriage, practiced food taboos, did not increase meal frequency, and did not have nutritional counseling. However, noticeably, there were no significant associations among the socio-demographic variables such as income and education. The multivariate multilevel mixed-effects logistic regression analysis showed that having two or more pregnancies, a history of miscarriage, the practice of food taboos, and nutritional counseling were significantly associated with undernutrition among pregnant women.

### Specific subgroups with high prevalence of undernutrition

We investigated the prevalence in different specific subgroups to demonstrate the consequences of these associations and to be more informative for healthcare. Based on the multivariate multilevel mixed-effect logistic regression model, we investigated the impact of having a combination of the significant variables on the prevalence of undernutrition. This showed that the prevalence of undernutrition was as high as 95% among women who had combination of identified risk factors (two or more pregnancies, history of miscarriage, practice of food taboos, and not getting nutritional counseling). These women were seven times more likely to be undernourished compared to those who did not have this combination of risk factors (OR: 6.95; 95% CI (0.80–60.41)). The undernutrition was nearly 60% among pregnant women who practice food taboo, and it was 70% with the women who had repeated pregnancies and history of miscarriage. It seemed that the reproductive health-related variables had more impact on undernutrition than the nutrition-related variables (Table 4).

**Table 4:** Nutritional status of the pregnant women with multiple risk factors, Sidama, Ethiopia, June 2019

Variables	Nutritional status of pregnant women		COR (95%CI)	AOR (95%CI)
	Normal	Undernutrition		
*Reproductive related predictors*				
Yes	19 (32%)	40 (68%)	4.05 (2.38–6.92)*	2.77 (1.41–5.43)
No	310 (66%)	161 (34%)	1	1
*Nutritional related predictors*				
Yes	49 (44%)	63 (56%)	2.61 (1.31–5.19)*	2.11 (1.32–3.35)
No	280 (67%)	138 (33%)	1	1
*Combination of all risk factors*				
Yes	1 (5%)	18 (95%)	32.28 (2.85–364.45)*	6.95 (0.80–60.41)
No	328 (64%)	183 (36%)	1	

Reproductive significant risk factors were having two or more pregnancy and history of miscarriage,

Nutrition related significant risk factors were food taboos and nutritional counseling

Combination of all identified risk factors were having two or more pregnancy, history of miscarriage, food *taboos and nutritional counseling*

*indicates that the variable has association

## Discussion

In this study, the prevalence of undernutrition among the study participants was 38%. Avoiding certain nutritious foods during pregnancy was common, often because women feared delivering a big baby. Having two or more pregnancies, food taboo practices, a history of miscarriage, and not getting nutrition counseling were associated with the prevalence of undernutrition during pregnancy. As a result, the prevalence was almost 70% among women who had two or more pregnancies and history of miscarriage, and close to 60% among women who practiced deliberate food restrictions during pregnancy. When a woman had both reproductive health-related risk factors and poor food practices, she was almost certainly undernourished.

The observed prevalence of undernutrition in our study is almost the same as in previous studies conducted in other parts of Ethiopia, such as Aleta Chuko (39%) [32], Miesso district of Oromia region (39%) [24], and Tigray region (41%) [18]. Conversely, the prevalence of our study is higher compared to studies conducted in Gondar (14%) [5], Dessie district (19%) [15], Silte Zone (22%) [16], Almata Hospital (22%) [8], Reyitu (Oromia region) (24%) [17], Halaba zone (27%) [33], Gambella (28%) [13], Central rift valley (32%) [7], and in the Afar region of Ethiopia (33%) [34]. In addition, our prevalence is high compared to the findings of studies conducted in Ashanti region of Ghana (11%) [4], findings of the systematic review from Africa (20%) [2], from a study conducted in rural Bangladesh (20%) [22], and compared to the global data of maternal undernutrition [12]. The observed differences between studies might be differences in set up and study population, as we included only second and third trimester pregnant women (advanced pregnancy deplete maternal nutritional reserve) [22] while other studies often included all term pregnant women. In general, it appears that the prevalence is mainly affected by local living conditions and traditions.

In this study, having two or more pregnancies was a risk factor for undernutrition among pregnant women, which is in line with findings from other studies conducted in other parts of the country [35, 36]. Possible reasons for this could be that the mother uses stored fat during pregnancy and subsequent lactation, and repeated pregnancy might put her at the increased risk of depleting those reserves [22]. Moreover, repeated pregnancies could result in preterm birth, low-birth weight, and short- and long term maternal complications, including undernutrition. Hence, our findings imply that having specific family planning interventions for those mothers who seem to have frequent pregnancies could increase the inter-pregnancy intervals. Furthermore, postpartum contraception use has a significant impact on increasing the inter-pregnancy interval, which in turn reduces unwanted pregnancies. [37]

The high risk of undernutrition among pregnant women who practice food taboo during pregnancy is consistent with the findings of studies done in other parts of Ethiopia, such as Afar [38], Hadya [39], and Addis Ababa [40]. Similarly, studies from sub-Saharan Africa such as Nigeria [26], Kenya [21], Sudan [41], and Gambia [42], reported the observation of food taboos during pregnancy as one of the factors contributing to maternal malnutrition. Additionally, according to the food-care health conceptual framework (UNICEF, 1990), food taboos and beliefs are considered to be ideological factors, among the basic causes of malnutrition [43]. Furthermore, avoiding foods has a negative effect on the health of the mother and newborn. Therefore, planning health education to reduce food taboos in the community might have an effect on minimizing maternal undernutrition. [44]

Consistent with the study findings from Haramaya and Dessie districts of Ethiopia, our study showed that women who did not get any nutritional counseling during pregnancy had a higher risk of undernutrition [15, 29]. A systematic review conducted on the effects of nutritional education and counseling on the nutritional status of pregnant women revealed a significant association supporting the finding of our current study [45]. Similarly, a study from Iran reported that nutritional counseling has a positive effect on the nutritional status of mothers [46]. The likely explanation for this is that counseling about the benefits of healthy eating and the effects of undernutrition could minimize the misconceptions of women about food during pregnancy. Furthermore, the WHO proclaimed that nutritional counseling is one of the strategies to improve the nutritional status of pregnant women [47]. As a result, integrating structured nutritional counseling and education into the existing maternal services could change the feeding habits of the pregnant women. [45, 48, 49]

In our study, women with a history of miscarriage were more at risk of undernutrition than their counterparts, which is in line with evidence from the Ethiopian demographic and health survey [50]. Another study conducted in the northern parts of the country found that a history of abortion was significantly related to maternal undernutrition [51]. A study from Nigeria showed that women who undergo an abortion lose essential micronutrients [51]. The possible justification could be that blood loss and associated stress during miscarriage could affect the health of women, including their nutritional status. We also demonstrated that preconception undernutrition is a risk factor for miscarriage, possibly creating a vicious cycle. In our view, one of the main aims of preconception services should be to detect undernutrition and provide counseling about proper nutrition before entering pregnancy.

In our study, women with multiple risk factors—having two or more pregnancies, history of miscarriage, the practice of food taboos, and not getting nutritional counseling—were seven times more at risk of undernutrition compared to those who did not have such a combination of risk factors. A systematic review also showed that nutritional and reproductive related factors were important determinants of the nutritional status of pregnant women [2]. In support of our findings, some studies also concluded that the most common risk factors for maternal undernutrition can be categorized as biological, behavioral, and societal [52]. As a result, raising awareness among healthcare workers about the risk factors for maternal undernutrition and having additional discussions about how to deal with highly prevalent issues is a critical recommendation. Furthermore, to reduce or possibly avoid such risks in the community, healthcare workers at each level of the maternal care unit should assess for nutritional conditions and counsel for a balanced diet as part of routine maternal healthcare services.

As a limitations, the cross-sectional nature of the study makes it impossible to say something about causal relationships between the risk factors and undernutrition. Therefore, longitudinal and, preferably, intervention studies should show to what extent changing the risk factors contributes to reducing undernutrition. We might also have over-fitted [53] our model by creating the subgroups based on a combination of risk factors. This gave us a very clear picture of how large the prevalence in certain subgroups could be and imagine the impact on healthcare. The way to deal with this over-fitting bias is to validate our results in future studies.

## Conclusion

Undernutrition is a highly prevalent problem among rural Ethiopian pregnant women, especially with those who avoid food and do not get counseled, having had two or more pregnancies and a history of miscarriage. Accordingly, strengthening health extension workers and other health professionals to focus on nutritional counseling and the risks of food taboos could be a potential way to improve undernutrition among pregnant women. Healthcare providers in the ANC and child service unit of the primary level of care, in particular, should be aware of the risks of maternal undernutrition and be prepared to address them. Moreover, it is evidenced that nutrition counseling integration into existing maternal and child service units is not well practiced. As a result, healthcare policymakers should reinforce nutritional education and counseling in routine maternal and child health services to address risks that contribute to maternal malnutrition.

## Data Availability

The data that support the findings of this study are available on request from the corresponding author via cheanana2007@gmail.com.

## Abbreviations

AIC: Akaike’s information criterion
AOR: Adjusted odds ratio
ANC: Antenatal care
BMI: Body mass index
BSc: Bachelor of Science
CI: Confidence interval
COR: Crude odds ratio
ETB: Ethiopian Birr
ICC: Intra-class correlation coefficient
IRB: Institutional Review Board
IUGR: Intra uterine growth restriction
MOH: Minister of Health
MUAC: Mid-upper arm circumference
SD: Standard deviation
SPSS: Software package of socially science
UNICEF: United Nations Children’s Fund
VIF: Variance inflation factor
WHO: World Health Organization
